# Taxonomic status and behavioural documentation of the troglobiont *Lithobiusmatulici* (Myriapoda, Chilopoda) from the Dinaric Alps: Are there semiaquatic centipedes in caves?

**DOI:** 10.3897/zookeys.848.33084

**Published:** 2019-05-20

**Authors:** László Dányi, Gergely Balázs, Ivan Hadrián Tuf

**Affiliations:** 1 Department of Zoology, Hungarian Natural History Museum, Baross u. 13, H-1088 Budapest, Hungary Hungarian Natural History Museum Budapest Hungary; 2 Department of Systematic Zoology and Ecology, Eötvös Loránd University, Pázmány Péter sétány 1/C, H-1117 Budapest, Hungary Palacký University Olomouc Budapest Hungary; 3 Department of Ecology and Environmental Sciences, Faculty of Science, Palacký University Olomouc, Šlechtitelů 27, CZ-77900 Olomouc, Czech Republic Palacký University Olomouc Olomouc Czech Republic

**Keywords:** Balkan Peninsula, biospeleology, cave, Lithobiomorpha, redescription, semiaquatic lifestyle, synonymy

## Abstract

*Lithobiusmatulici* Verhoeff, 1899 is redescribed based on type material and newly collected specimens. *Strandiolusjugoslavicus* Hoffer, 1937, described from another cave in the same region in Bosnia and Hercegovina, is presented as a junior subjective synonym of *L.matulici* (syn. nov.). *L.matulici* is shown to be most closely related to *Lithobiusremyi* Jawłowski, 1933, type species of the subgenus Thracolithobius Matic, 1962. The completeness of the chitin-lines on the forcipular coxosternite is discussed as a promising character for interspecific differentiation within Lithobiomorpha. Documentation of hitherto unknown semiaquatic behaviour in *L.matulici* and other cave-dwelling centipede species from Herzegovinian-, Montenegrin- and Pyrenean caves is presented.

## Introduction

**Figure 1.**
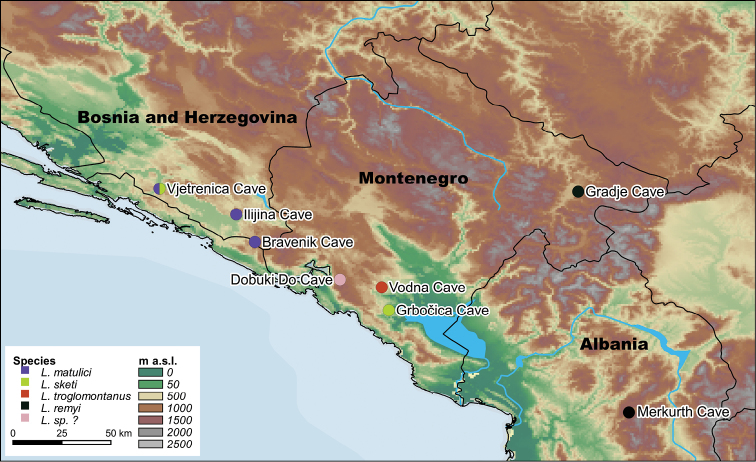
Occurrences of blind *Lithobius* species in the South Dinaric Alps.

Many species of lithobiomorph centipedes have been described from European caves during the 19^th^ and 20^th^ centuries (e.g. Verhoeff 1899; Matic and Dărăbanţu 1968), as well as more recently (e.g. Negrea and Minelli 1994; Iorio 2009, 2015; Stoev et al. 2013; Akkari et al. 2017). The degree of cave adaptation in the morphology of these species is rather variable: while some of them still have ocelli and rather short appendages similar to those in epigeic species, other taxa present highly troglomorphic characters, such as being completely blind and having strongly elongated legs and antennae (Folkmanová 1940; Lewis 1981). Regarding the Dinaric Mountains on the Balkan Peninsula and considering only the species with functionally articulated tarsi, five species variously placed in six genera/subgenera have been described as belonging to the latter, troglomorphic group: Lithobius (Oligobothrus) matulicii [sic] Verhoeff, 1899; *Strandiolusjugoslavicus* Hoffer, 1937; *Mesobothrustroglomontanus* Folkmanová, 1940; Lithobius (Troglolithobius) sketi Matic & Dărăbanţu, 1968; and Lithobius (Thracolithobius) remyi Jawłowski, 1933. In addition to their troglomorphic features, all of these taxa might be considered as troglobionts according to the definition of Sket (2008), as they have only been found in caves and never in surface (epigean) habitats. Most of these species are known only from their original description and only from their one or two type locality cave(s) in South Herzegovina, Montenegro, and North Albania (Fig. 1). When revising the taxonomy of the above mentioned (sub)genera, Stoev (1997) concluded that probably none of these are natural taxa and synonymised *Strandiolus* Hoffer, 1937, *Hemibothrus* Folkmanová, 1946 (replacement name for *Mesobothrus* Folkmanová, 1940 due to homonymy) and *Troglolithobius* Matic, 1967 under *Lithobius* Leach, 1814 (s.s.). Regarding *L.matulici*, *S.jugoslavicus*, *M.troglomontanus*, and *L.sketi* he stated that: “It will be no great surprise if the four Balkan „species” are in fact highly variable cave populations of one or two species. Only additional collecting and/or type revision can settle this problem.” (Stoev 1997: 90).

Just as suggested more than 20 years ago, freshly collected specimens from that area combined with the study of type material allowed us to revise one of these species, *L.matulici*, and to show that one of the others, *S.jugoslavicus*, is its junior subjective synonym.

Some morphological and behavioural characters not highlighted in earlier descriptions are discussed here in detail:

1. The posteriorly rounded form of the 14^th^ tergite might indicate a close relation of *L.matulici* to members of the subgenus Thracolithobius Matic, 1962 (Zapparoli and Edgecombe 2011);

2. The completeness of the chitin-line on the forcipular coxosternite is an important specific character within several genera in Geophilomorpha (Bonato et al. 2011), but until now, it has not been used in Lithobiomorpha. Our unpublished preliminary studies show that this character is also probably useful for interspecific differentiation in this group, as it seems to have different character states (i.e. incomplete, or complete – as in *L.matulici*) which are stable within species;

3. An amphibious lifestyle in freshwater has not been reported for lithobiomorph centipedes yet, and there is only one species with such behaviour within Chilopoda as a whole. Documentation of underwater activity in cave-dwelling species is presented here, from which at least one is ascertained to be *L.matulici*; another observation made in a Pyrenean cave indicates that this behaviour might be actually rather widespread among cave-dwelling centipedes, similarly as in troglobiont millipedes, where a few amphibious species are already known (Enghoff 1985).

## Material and methods

For light microscopy, specimens from Bravenik Cave (Bosnia and Herzegovina, Grab (near Trebinje), 42°35.97'N, 18°25.29'E) were cleared in a mixture of lactic acid and glycerol (3:1) on temporary slides. Two specimens were later cleared also in potassium-hydroxide and mounted in Euparal on permanent slides (all deposited in the Myriapoda Collection of the Hungarian Natural History Museum, Budapest, Hungary: inventory numbers HNHM chilopr-377–378; HNHM chilo-6330). Slides were examined under a Leica DM 1000 microscope equipped with a drawing tube for preparing line drawings. The map for Figure 1 was generated with QGIS version 3.2.2. (QGIS Development Team 2018).

Terminology for external anatomy follows Bonato et al. (2010).

The following abbreviations are used in the text and tables: a—anterior, C—coxa, D—dorsal, F—femur, m—median, p—posterior, P—prefemur, T—tibia, t—trochanter, V—ventral.

## Taxonomic part

### Class Chilopoda Latreille, 1817

#### Order Lithobiomorpha Pocock, 1895

##### Family Lithobiidae Newport, 1844

###### Subfamily Lithobiinae Newport, 1844

####### Genus *Lithobius* Leach, 1814

######## Lithobius (Lithobius) matulici

Taxon classificationAnimaliaLithobiomorphaLithobiidae

Verhoeff, 1899

Lithobius (Oligobothrus) Matulicii [sic] Verhoeff 1899: 452, figs II, III, V (original description)
Lithobius (Oligobothrus) Matulicii [sic] Verhoeff: Verhoeff 1900: 158, 167 (in key; new data)
Lithobius (Lithobius) matulicii [sic] Verhoeff: Verhoeff 1937: 196 (in key); Matic 1960: 447 (in key)
Lithobius (Troglolithobius) matulicii [sic] Verhoeff: Matic 1967: 90 (erection of the new subgenus Troglolithobius); Matic and Dărăbanţu 1968: 211, figs 4a–4g, tab. 4 (redescription); Lewis 1981: 106 (mentions enlarged Tömösváry organ); Kos 1992: 357 (in list)
Lithobius (s.s.) matulici Verhoeff: Folkmanová 1946: 64 (in key, emendation); Stoev 1997: 90 (synonymisation of Troglolithobius); Zapparoli and Edgecombe 2011: 377 (only mentions)
Strandiolus jugoslavicus Hoffer 1937: 429, figs 1–10 (syn. nov.) (original description, erection of new genus); Jeekel 2005: 31 (in list)
Lithobius jugoslavicus (Hoffer): Stoev 1997: 90 (synonymisation of Strandiolus)

######### Remark on the origin of name.

The species was dedicated to Lucijan von Matulić (teacher at a high school in Trebinje and founder of the first Speleological Society in Bosnia and Herzegovina in Trebinje in 1911), thus it was emended to “*matulici*” by Folkmanová (1946).

######### Type locality.

Ilijina Pećina (as “Elias Höhle bei Trebinje” in the original description (Verhoeff 1899)) 42°43.63'N, 18°20.17'E. (Type locality of *S.jugoslavicus*: Vjetrenica Cave – as “grotte sur le mont ‘Brencovac’ près de Zavala en Popovo polje” in the original description (Hoffer 1937), 42°50.752'N, 17°59.028'E).

######### Material examined.

**Figures 2, 3.**
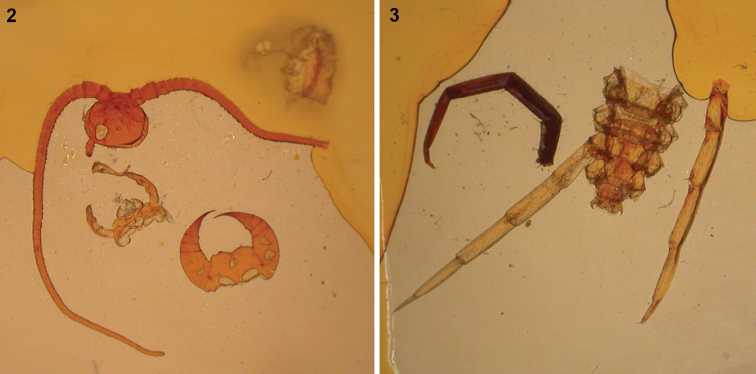
Holotype of *Lithobiusmatulici* Verhoeff, 1899 on slides from the Museum für Naturkunde, Berlin **2** slide No. 266 **3** slide No. 267.

**Type material**: female holotype on two slides (Slide No. 266 and 267) housed by the Museum für Naturkunde, Berlin. The slides were mounted in Canada balsam, but in an inappropriate way since they are partially dried out (Figs 2, 3). Such drying may probably happen because of the mixing of the Canada balsam with a diluting-agent, like glow-oil or xylene, at a too high of a level.

Slide No. 266: cephalic capsule, mandibles, maxillae, forcipules and forcipular tergite, half of the 1^st^ leg-bearing segment’s tergite (Fig. 2).

Slide No. 267: posterior part of body from 12^th^ segment, legs missing except right 14^th^ leg and the 15^th^ pair detached. Right ultimate leg was probably not macerated in any clearing agents before slide mounting, since the muscles are well visible inside (Fig. 3). All the other parts cleared, probably via potassium hydroxide, because their muscles were dissolved.

2 ♀ (HNHM chilo-6330, HNHM chilopr-377), 1 subadult ♀ (HNHM chilopr-378): Bosnia and Herzegovina, Bravenik Cave, Grab (near Trebinje), 42°35.97'N, 18°25.29'E, 20.07–20.09.2008, leg. Roman Lohaj.

######### Further data.

A subadult female of 12 mm from the type locality cave (Verhoeff 1900; not studied). Two males and three females from the Vjetrenica Cave (type locality of male *Strandiolusjugoslavicus*) (Matic and Dărăbanţu 1968; not studied).

######### Diagnosis.

A Lithobius Leach, 1814 species (subgenus Lithobius Leach, 1814) of a length about 14–26 mm; with long antennae of 76–110 articles, reaching the posterior end of tergites 8–9 when folded backwards; ocelli absent; Tömösváry’s organ large, with a diameter 0.08–0.1 times of the length of the cephalic plate; 2+2–3+4 obtuse and short teeth on dental margin of forcipular coxosternum, porodonts large, about 2.8–3 times longer and 1.3–2 times broader than teeth; chitin-lines on the forcipular coxosternite reaching the posterior margin of coxosternite; posterior part of 14^th^ tergite without setae-bearing area in both sexes; legs 1–13 with long anterior and posterior accessory spines; 14^th^ and 15^th^ pairs of legs without accessory spines, without secondary sexual characters, and with the following plectrotaxy 15: -,-,(m)p,-,-/-m,mp,m,- and 14: -,-,(m)p,-,-/-m,mp,m,-; 3,4,4,3–5,5,5,5 coxal pores arranged in a single row; female gonopods with 2+2 spurs on first article, gonopodal claw bipartite.

######### Redescription based on material examined and on literature.

Where differences between specimens from different caves occur, they are highlighted at the given characters.

**Figures 4–17.**
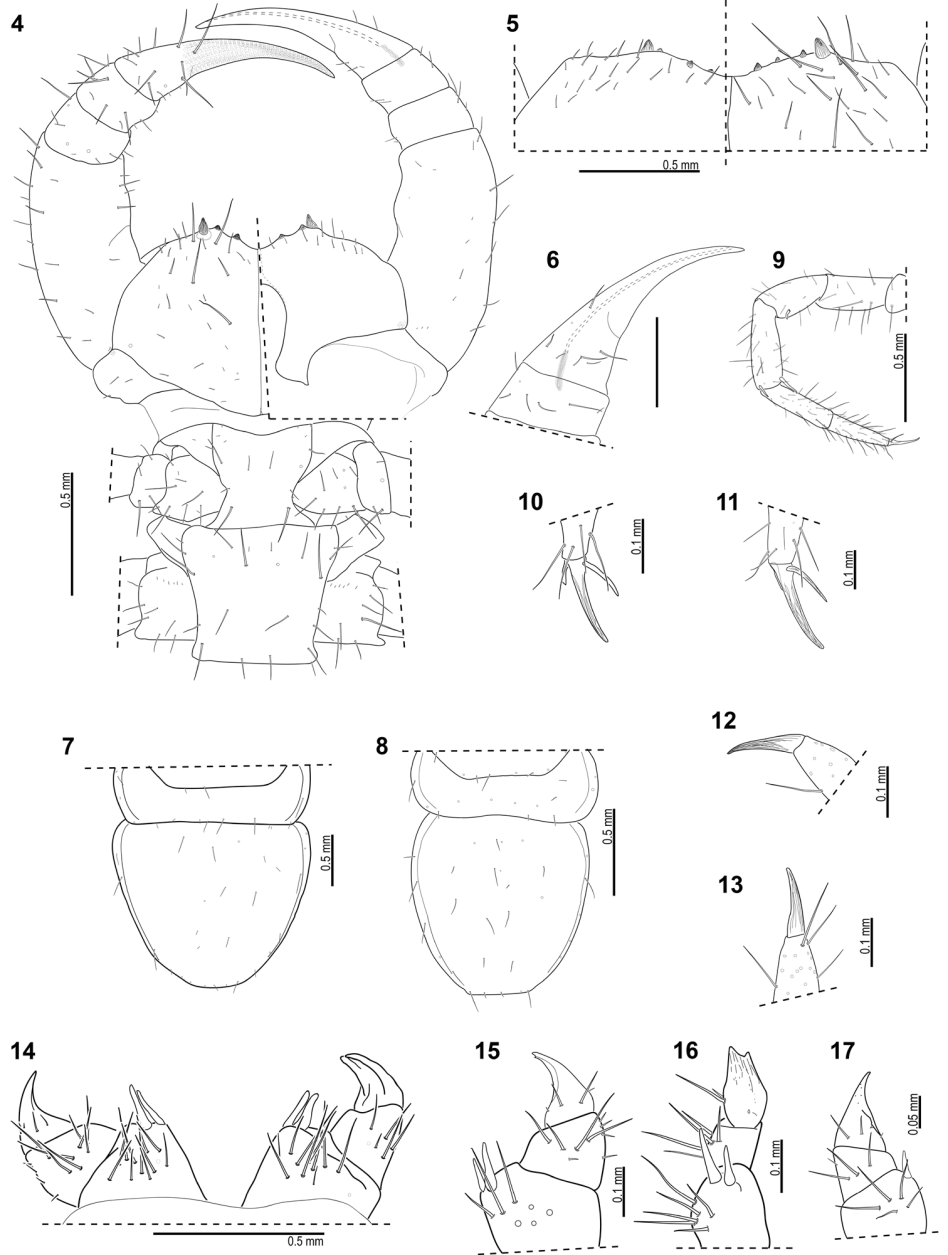
*Lithobiusmatulici* Verhoeff, 1899 (holotype 5–7, 14; HNHM chilopr-377 4, 8–13, 15–16; HNHM chilopr-378 17) **4** forcipules and trunk segments 1–2, left side of forcipules with ventral view, right side with dorsal view **5** coxosternal dentation, left side with dorsal view, right side with ventral view **6** tarsungulum and forcipular tibia of the holotype (ventral view) **7–8** tergites 13–14 **9** right leg 1 (anterior view) **10** claw of right leg 1 (anterior view) **11** claw of right leg 13 (anterior view) **12** claw of right leg 14 (posteriomedial view) **13** claw of left leg 15 (posteromedial view) **14** gonopods of holotype **15** female gonopod (lateral view) **16** female gonopod (anterior view) **17** subadult female gonopod (right, lateral view).

Body length 14–26 mm (holotype 21.5 mm according to the original description; specimens from Vjetrenica Cave 20–26 mm (26 mm in holotype of *S.jugoslavicus*), specimens from Bravenik Cave 14–17 mm). Coloration yellowish-white in alcohol. The whole cuticle is thin and rather soft, almost transparent, wrinkled on the cephalic plate and tergites (wrinkling not mentioned for specimens from Vjetrenica Cave). Cephalic plate, forcipules and body without punctae. Cephalic plate as broad as tergite 8, about as broad as long (1.96 mm long and 2.28 mm wide in holotype, but width obviously affected there by flattening at slide-mounting; Fig. 2). Ocelli missing. Tömösváry’s organ very large, with diameter 0.08–0.1 times of the length of the cephalic plate, placed on the ventral to anterolateral margin of cephalic pleurite. Antennae composed of 76–110 articles (in holotype right antenna with 106 articles, left antenna broken and distal part missing; 85–88 articles in holotype of *S.jugoslavicus* and 106–110 in other specimens from Vjetrenica Cave), long (7.8–18.5 mm, 13.5 mm in holotype, 18.5 mm in holotype of *S.jugoslavicus*), reaching the posterior end of tergites 8–9. Most articles short, probably from secondary segmentation, with only one whorl of setae (number of whorl of setae not documented in specimens from Vjetrenica Cave but proportion of antennal articles illustrated as the same in Hoffer 1937: fig. 1). Forcipular coxosternite broad, with 2+2–3+4 obtuse and short teeth (usually 3+3 as in the holotype (Fig. 5), in the holotype of *S.jugoslavicus* and four other specimens from Vjetrenica Cave, and in the specimen HNHM chilo-6330 from Bravenik Cave; 3+4 in only one specimen from Vjetrenica Cave according to Matic and Dărăbanţu (1968: fig. 4c), 2+2 in specimens HNHM chilopr-377–378 (Fig. 4) from Bravenik Cave; porodonts stout and strong, about 2.8–3 times longer and 1.3–2 times broader than teeth; dentate part of the coxosternite concave, shoulder of coxosternite broad (Figs 4, 5); chitin-lines reaching the posterior margin of coxosternite (Fig. 4). Lateral edges of trochanteroprefemur and part of coxosternite extended beyond cephalic plate. Calyx of poison gland 6.5–7 times as long as wide, about ¼ situated in distal half of forcipular tibia (Figs 4, 6) (not known for specimens from the Vjetrenica Cave). Forcipular tergite narrower than cephalic plate with a ratio of about 0.8 (in holotype of *S.jugoslavicus* similar ratio according to Hoffer 1937: fig. 1, but about 1.1 in his fig. 8; 0.85 for another specimen from the same Vjetrenica Cave according to Matic and Dărăbanţu (1968: fig. 4b)). Lateral sides of body rather parallel, only slightly broadened at tergites 8–10. Tergites 3, 5, 8, 10, 12 and 14 posteriorly rounded, without protuberances; posterior end of tergite 14 semicircular (less pronounced in younger specimens from Bravenik Cave (Fig. 8), almost perfect in the female holotype (Fig. 7) and in the male holotype of *S.jugoslavicus* illustrated by Hoffer (1937: fig. 1)). Sternites 1–10 longer than broad, sternites 11–15 shorter than broad (sternites 1–11 missing and not documented in holotype). Sternite 15 in female trapeziform, posterolaterally narrower than anterolaterally, with straight posterior border, in male longer than broad according to Hoffer (1937: fig. 10, from Vjetrenica Cave, not documented from other caves). Legs elongated, 14–15^th^ without modifications. Length of leg articles of holotype (in mm): leg 14: trochanter+prefemur = 1.7, femur = 2.0, tibia = 2.2, tarsus 1 = 2.0, tarsus 2 = 0.8; legs 15: trochanter+prefemur = 1.6–1.7, femur = 2.1–2.2, tibia = 2.2–2.4, tarsus 1 = 2.0–2.1, tarsus 2 = 0.8–0.9. Right ultimate leg of holotype with tarsus 2 having an ‘articulated’ appearance (Fig. 3), although only collapsed as an artefact (probably caused during the mounting). Leg plectrotaxy as in Tables 1–3 (differences between cave populations given in footnotes), spines 1–6VmF and 1VmT missing in the subadult female of ~11 mm (HNHM chilopr-378). Legs 14–15 with claws of usual proportions, without accessory spines (Figs 12, 13); legs 1–13 with elongated claws and with elongated anterior and posterior accessory spines (Figs 10, 11), relative length of accessory spines highest on legs 11–12: about 0.5 of claw’s length for the anterior and 0.3 for the posterior spine (from Vjetrenica Cave Hoffer (1937: fig. 6) illustrated for leg 13 ratios of about 0.4 in both spines, while Matic and Dărăbanţu (1968: fig. 4g) illustrated for leg 10 ratios of 0.8 and 0.2). 3,4,4,3–5,5,5,5 coxal pores arranged in one line. In the original description Verhoeff (1899) mentioned 2(+1),3,4,3 as number for coxal pores in the holotype, but in fact it is 4,4,4,3 on legs 12–15 respectively; in *S.jugoslavicus* only legs 14–15 were documented with 5 and 4 coxal pores respectively (Hoffer 1937: fig. 10; in the text erroneously mentioned 4 and 5 respectively, which would be an unusual pattern in Lithobiomorpha). For the specimens from the same Vjetrenica Cave Matic and Dărăbanţu (1968) mentioned 5,5,5,5 coxal pores, while in the specimens from Bravenik Cave we found 3,4,4(5),3(4).

**Table 1.** *Lithobiusmatulici* Verhoeff, 1899. Plectrotaxy of holotype, legs 1–13 missing.

**Leg pairs**	**Ventral**					**Dorsal**			
	**C**	**t**	**P**	**F**	**T**	**C**	**P**	**F**	**T**
14–15	–	m	mp	m	–	–	mp	–	–

**Table 2.** *Lithobiusmatulici* Verhoeff, 1899. Plectrotaxy of a young female (HNHM chilopr-377) from Bravenik Cave, Grab (near Trebinje), Bosnia and Herzegovina (brackets indicate spines present asymmetrically).

**Leg pairs**	**Ventral**					**Dorsal**			
	**C**	**t**	**P**	**F**	**T**	**C**	**P**	**F**	**T**
1–12	–	–	–	m	m	–	–	–	a
13	–	m	mp	m(p)	m	–	p	–	a
14–15	–	m	mp	m	–	–	mp	–	–

**Table 3.** *Lithobiusmatulici* Verhoeff, 1899. Plectrotaxy of adults combined from all available data (brackets indicate spines missing in some cases).

**Leg pairs**	**Ventral**					**Dorsal**			
	**C**	**t**	**P**	**F**	**T**	**C**	**P**	**F**	**T**
1	–	–	–	(m)^†^	m	–	–	–	a
2–11	–	–	–	m	m	–	–	–	a
12	–	–	(mp)^‡^	m(p)^‡^	m	–	–	–	a
13	–	(m)^§^	(mp)^‡^	m(p)^‡^	(m)^†^	–	(p)^†^	–^|^	a
14	–	m	mp^¶^	m^¶^	–^¶^	–	(m)^‡^p	–^|^	–
15	–	m	mp	m	–	–	(m)(p)^#^	–^|^	–

^†^Absent in *S.jugoslavicus* according to Hoffman (1937), but present in specimens from the same cave according to Matic and Dărăbanţu (1968: table 3). ^‡^Present in *S.jugoslavicus* according to Hoffman (1937), but absent in specimens from the same cave according to Matic and Dărăbanţu (1968: table 3). ^§^Present in only one specimen from Bravenik Cave (see Table 2). ^|^The presence of spines on femora instead of prefemora in Matic and Dărăbanţu (1968: table 3) is most probably a typing or printing error, i.e. marking the spines in the wrong column of the table. ^¶^The ventral plectrotaxy given for leg 14 by Matic and Dărăbanţu (1968: tab. 3), -,m,m,mp,m, i.e. more spines on femur than on prefemur is very unusual in *Lithobius*, thus a printing error in the table might be suspected. ^#^Only spine “p” present in specimens from the Vjetrenica Cave according to Matic and Dărăbanţu (1968: table 3). Only one spine in *S.jugoslavicus* from the same cave according to Hoffman’s (1937) plectrotaxy table, which is spine “m” according to the illustration in the same work (Hoffman 1937: fig. 1). Both spines “p” and “m” present in the holotype of *L.matulici* and in the specimens from Bravenik Cave.

Female first genital sternite longer than wide, with 22–40 evenly scattered setae (40 in holotype; not known in specimens from Vjetrenica Cave); posterior border almost straight (Fig. 14) (not known in specimens from Vjetrenica Cave). Female gonopods with thin setae and 2+2 elongated spurs on first article (holotype in Fig. 14; unequal spurs in younger adults as in Figs 15, 16; 1+1 in a subadult specimen in Fig. 17). Lateral side of female gonopods with 7–12 moderate to long setae on first article, 5–8 setae on second and 1 or 2 setae on third article, arranged as in Figures 14–16 (only 4 setae on first article in a specimen from Vjetrenica Cave according to Matic and Dărăbanţu (1968: fig. 4d) but their drawing is probably inaccurate in this detail); dorsal side of gonopod with about 4 weak spines on second article and 1–3 minute spines on third article (Figs 14–16), medial side of female gonopods without setae (not known in specimens from Vjetrenica Cave). Gonopodal claw bipartite (on left gonopod of holotype (Fig. 14) misinterpreted by Verhoeff (1899: fig. V) as tripartite); medial tip smaller than lateral (Fig. 16).

######### Remarks on synonymy.

*Strandiolusjugoslavicus* was described by Hoffer (1937) on a single male specimen from the Vjetrenica Cave (“grotte sur le mont ‘Brencovac’ près de Zavala en Popovo polje”, 42°50.752'N 17°59.028'E) without comparison with *Lithobiusmatulici* Verhoeff, 1899, known from another cave only about 32 km away. The depository of the type is unknown, and it was not found at the National Museum in Prague (Dolejš 2015) where that part of Hoffer’s material is housed that we know to exist. However, the original description is very detailed, supplemented with illustrations, and fits in every important character with Verhoeff’s original description, but also with the holotype of *matulici*, as well as the fresh material studied by us. It also fits the five topotypic specimens described by Matic and Dărăbanţu (1968). The fact that Matic and Dărăbanţu (1968) identified these topotypic specimens as *L.matulici* (without any notes on *S.jugoslavicus*) also supports our conclusion that *S.jugoslavicus* is a subjective junior synonym of *L.matulici* (syn. nov.). Because also neighbouring caves might be completely isolated from each other, high-level genetic separation of cave populations might occur even in cases where no morphological differences of the specimens are obvious. Future molecular studies might easily support our decision based on morphology.

######### Taxonomic remarks.

The posteriorly semicircular form of the 14^th^ tergite has not been highlighted for this species by the earlier authors, although it was illustrated by Hoffer’s (1937: fig. 1) drawing on the habitus of the holotype of *S.jugoslavicus* and Verhoeff (1899) mentioned that the posterior corners of the tergites 3, 5, 8, 10, 12, and 14 are exceptionally strongly rounded. It is present in the holotype of *matulici* (Fig. 7) and in our fresh specimens as well. Matic and Dărăbanţu (1968) seem to have overlooked this character, as they only mentioned that the tergites are without posterior triangular projections. Hoffer (1937) characterised the tergites as of oval in shape, but for more details he referred to his drawing with the holotype which depicts tergite 14 with rounded posterior margin.

The shape of the 14^th^ tergite seems to indicate a close relation of *L.matulici* to the members of the subgenus Thracolithobius Matic, 1962 (Zapparoli and Edgecombe 2011), especially to its type species, *Lithobiusremyi*, described from the Gradje Cave (Montenegro), which is only 95–150 km from the known occurrences of *L.matulici*, and also reported from the North Albanian Merkurth Cave (Stoev 1996). As the posteriorly semicircular form of the 14^th^ tergite is the key character defining *Thracolithobius*, we could consider *L.matulici* as member of this subgenus, but we refrain to do for reasons of nomenclatural stability. Including *L.matulici* in *Thracolithobius* would result in a situation in which the generic name *Strandiolus* Hoffer, 1937 would became a subjective senior synonym of *Thracolithobius* Matic, 1962 according to the principle of priority (ICZN 1999: Art. 23), because its type species, *Strandiolusjugoslavicus* Hoffer, 1937, is synonymised in the present paper under *L.matulici* (see above). *Strandiolus* was synonymised under *Lithobius* (s.s.) by Stoev (1997) (also proposed earlier informally and without explanation by Folkmanová (1946) in a key) because its differential characters are either actually common in *Lithobius* (s.s.) – three ‘claws’ on legs 1–13, reduced leg plectrotaxy, notched lateral edges of head, absence of tergal projections, form of maxillae II – or adaptations to the cave environment – absence of ocelli, elongation of legs and narrow anterior sternites, depigmentation, high number of antennal articles – and as such of no taxonomical importance. Meanwhile, *Thracolithobius* Matic, 1962 is considered as a valid subgenus (Stoev 1997; Shelley 2006; Ćurčić et al. 2008; Zapparoli and Edgecombe 2011) with three species – *L.dacicus* Matic, 1959, *L.inexpectatus* Matic, 1962, *L.remyi* Jawlowski, 1933 – but the monopyhly of this group might be questioned. The only common character defining this subgenus is the shape of the 14^th^ tergite, a character that however has already been proven to vary at the inter(sub)specific level in *Lithobius* (Andersson 1979) and in another lithobiomorph genus, *Eupolybothrus* (Stoev et al. 2013; Akkari et al. 2017). Apart from this character, the members of the subgenus seem to be rather different in several other features (e.g. presence/absence of ocelli and a wart-like structure on forcipular tarsungulum) and *L.matulici* differs actually from the members of *Thracolithobius* even in an aspect of the 14^th^ tergite: the rounded shape is present in *matulici* also in females, while it is known only from males in the other species. Although at least *L.remyi* and *L.matulici* seem to be similar also in some other features (lack of ocelli, strong porodonts, coxosternal dentation) this may be also due to convergent adaptation to a similar lifestyle in cave environments.

According to this, we can expect that molecular studies will prove *Thracolithobius* to be polyphyletic with its members spread among different clades of *Lithobius* (s.l.), which would result in its synonymisation under *Lithobius* (s.s.); and this would be the case again even if its name would be changed here to the older name *Strandiolus*. In case future molecular studies give an opposite result (i.e. monophyly of *Thracolithobius* including *L.matulici*), *Strandiolus* might be revalidated.

######### Differential diagnosis.

Among the *Lithobius* species with a posteriorly rounded tergite 14, *L.matulici* seems to be most similar to *L.remyi*, but differs from that species in size (11–13 mm in *remyi*, 14–26 mm in *matulici*), number of antennal articles (56–64 in *remyi*, 76–110 in *matulici*), and the shape of the female gonopodal claw (tripartite in *remyi*, bipartite in *matulici*). From *L.dacicus*, *L.matulici* differs in size (about 12 mm in *dacicus*, 14–26 mm in *matulici*), number of antennal articles (37–61 in *dacicus*, 76–110 in *matulici*), coxosternal dentation (2+2–3+4 small and obtuse teeth in *matulici*, 2+2 well developed teeth in *dacicus*), and completeness of coxosternal chitin-lines (not reaching the posterior margin of the coxosternite in *dacicus*, reaching it in *matulici*). *Lithobiusinexpectatus* is distinguished from *L.matulici* by having 12–14 ocelli (missing in *matulici*), by the coxosternal dentation (2+2–3+4 small and obtuse teeth and very strong porodonts in *matulici*, 2+2 larger teeth and slender porodonts in *inexpectatus*), the number of antennal articles (42 in *inexpectatus*, 76–110 in *matulici*), the presence of accessory spines on legs 14–15 (absent in *matulici*), the shape of the female gonopod claw (tripartite in *inexpectatus*, bipartite in *matulici*), and plectrotaxy (1–15VaF, 1–13VaT, 1–14VpT, 8–15DaP, 1–15DpP, 1–13DaF, 3–15DpF and 3–15DpT present in *inexpectatus*, missing in *matulici*).

Although no rounded form of tergite 14 is known for it, *L.sketi* was stated to be very similar to *L.matulici*, and they also co-occur in Vjetrenica Cave (Matic and Dărăbanţu 1968). The two species are readily distinguished by the accessory spines on the 14–15^th^ legs (present in *sketi*, missing in *matulici*), by the number and arrangement of coxal pores (5–9 per coxa arranged in 2 partly irregular rows in *sketi*, 3–5 per coxa in a single row in *matulici*), the female gonopods (1+1 spurs and simple claw in *sketi*, 2+2 spurs and bipartite claw in *matulici*), and their plectrotaxy (1–13VpP, 1–15DaP, 1–15DpP, 1–14DaF, 1–15DpF and 2–15DpT present in *sketi*, missing in *matulici*).

######### Semiaquatic behaviour.

**Figures 18–20.**
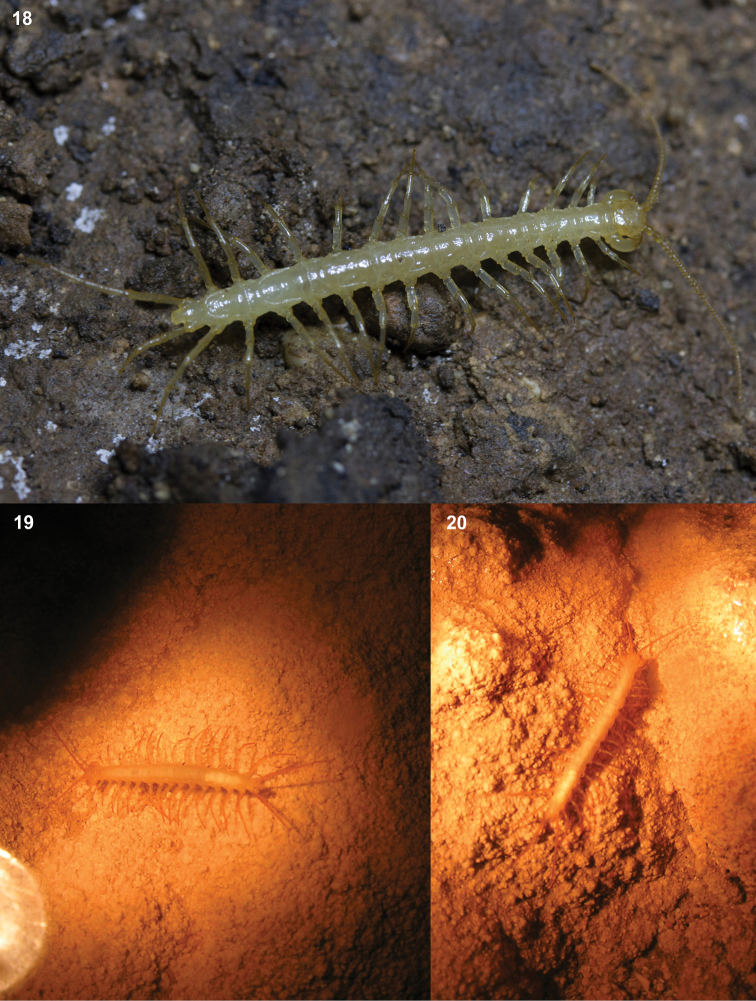
*Lithobius* specimens from Dinaric caves **18** living Lithobiuscf.matulici specimen of ca. 25 mm length from the Vjetrenica Cave (Bosnia and Herzegovina) (photo by Gergely Balázs) **19–20***Lithobius* sp. under water in the Dobuki Do Cave (Montenegro) (photos by Zsolt Polacsek).

One lithobiomorph specimen was found in July 2014 while one of the authors, G. Balázs, was diving in Vjetrenica Cave. The specimen was in a water-filled part of the cave (Donje Vjetrenica), freely and consciously walking on the underwater bottom at a depth of 3 metres, at a distance of about 30 metres from any terrestrial microhabitats (i.e. chambers with air). This specimen was without any signs of distress (no spasms, no enfeeblement). There was no flood in the cave at that time, the water was still (not flowing), and thus a simple flushing away of the specimen from the water’s edge might be ruled out. This individual spent another 2 hours in the water, while kept captured by the diver and escaped later during photographic documentation. In the photograph (Fig. 18), the 14^th^ tergite of the specimen seems clearly rounded posteriorly, and thus it can be considered as *L.matulici* with confidence. Similar cases of lithobiomorph specimens on the bottom of water (puddles) in caves were photo-documented in Montenegro (Dobuki Do: 42°25.739'N 18°48.716'E: August 2006, Zsolt Polacsek in litt., Figs 19, 20; July 2018, Márton Mede in litt.), and from a cave in North Spain (Tibia-Fresca Cave System, 19 July 2016, see supplementary file 1: Video 1; Zsolt Polacsek in litt.).

### Key for the Dinaric *Lithobius* species without ocelli:

**Table:** 

1	Tarsus 1–13 biarticulated	**2**
–	Tarsus 1–13 single	**4**
2	Claw of ultimate and penultimate legs simple, without accessory claw	**3**
–	Claw of ultimate and penultimate legs with accessory claw	**L. (Lithobius) sketi Matic & Dărăbanţu, 1968**
3	Number of antennal articles 62–64; female gonopodal claw tripartite; posterior half of tergite 14 in males with setaceous field and with or without a swelling	**L. (Thracolithobius) remyi Jawłowski, 1933**
–	Number of antennal articles 76–110; female gonopodal claw bipartite; posterior half of tergite 14 in males without setaceous field or swelling	**L. (Lithobius) matulici Verhoeff, 1899**
4	Antennae composed of 20 (21) or fewer articles	**L. (Monotarsobius) zveri (Matic & Stenzer, 1977)**
–	Antennae composed of more than 23 articles	**5**
5	Antennae composed of 30–38 articles	**L. (Sigibius) reiseri Verhoeff, 1900**
–	Antennae composed of 24–28 articles	**L. (Sigibius) apfelbecki Verhoeff, 1900**

## Discussion

**Chitin-line.** A suture extending posteromedially from the coxosternal condyle of the forcipule in lithobiomorphs corresponds in position to the chitin-line of geophilomorphs. These two structures are a little different in their construction in the two groups and are either a strongly sclerotised narrow stripe in Geophilomorpha or a weak suture in Lithobiomorpha according to Bonato et al. (2010). However, a weak suture is also present along the stripe in geophilomorphs, and weak sclerotisation is present along the suture also in lithobiomorphs (orig. obs.). Thus, the homology of the two structures seems probable, and we prefer to also use this established term (Bonato et al. 2010) in Lithobiomorpha, just as it has already been used by Latzel (1880).

While the chitin-line is an incomplete suture (i.e. not reaching the posterior margin of the coxosternite) in several lithobiomorph species, it is complete in *L.matulici*. Our preliminary unpublished studies reveal that a complete chitin-line is probably not rare at all (e.g. in *Lithobiusforficatus* (Linnaeus, 1758), *Lithobiusmicrops* Meinert, 1868, and *Lithobiusburzenlandicus* Verhoeff, 1931). The states of this character seem to be stable within species, as well as in specimens of different age which promises that it might be useful for some cases of interspecific differentiation.

**Semiaquatic behaviour.** Semiaquatic behaviour in terms of actively and regularly moving into the water has never before been reported for lithobiomorphs, but even for Myriapoda as a whole there have been few examples. In the following paragraphs a short overview is given (for Chilopoda as well as for millipedes), starting from observation of animals actively seeking water to species enduring inundation out of necessity in flood-prone areas.

Only two publications mention active semiaquatic behaviour in Chilopoda. One is the only report of centipedes entering freshwater on their own free will (Armitage 1982). This short paper reports on several specimens of the geophilomorph *Strigamiamaritima* (Leach, 1817) found on two occasions in a small stream in England, where they possibly entered the water to hunt for caddisfly larvae (Armitage 1982). The other case is of a scolopendromorph specimen which was possibly hunting underwater (Moraes and Chagas-Júnior 2009). The centipede was found dead in a sea anemone which had probably caught it under water.

A semiaquatic lifestyle is more frequently noted for millipedes. Some species have been reported from under stones in streams in France (Causard 1903) and Australia (Burrows et al. 1994), and one species in South America is known to be able to live submerged for several months in subadult stadia (Adis 1986). Three additional, possibly highly water-adapted species have been reported from Guyana and from widely dispersed Atlantic and Pacific islands (Golovatch and Kime 2009). From caves there are several millipede species described as semiaquatic, for example some julids and polydesmids in the Italo-Balkan region of Europe (Adis et al. 1997; Enghoff et al. 1997; Antić et al. 2017). These cave millipedes enter water on purpose, spend a long time submersed, and have modified mouthparts, which are probably adapted to filtering and screening suspended organic particles from the water (Adis et al. 1997). Similar mouthparts are also known from some other cave-dwelling millipede species from the Caucasus and Papua New Guinea (Enghoff 1985), suggesting that semiaquatic behaviour might be more common in diplopods than generally acknowledged.

Some observations show centipedes to choose swimming as a way of escape when attacked or disturbed. Zulka (1991) published the first observations of this for *Lithobiuscurtipes* (C.L. Koch, 1847) and *Lamyctesemarginatus* (Newport, 1844), which entered water from objects standing out of surrounding water when he tried to catch them. Even when there are terrestrial pathways for escape, some species or at least specimens chose water: Siriwut et al. (2016) mentioned an individual of *Scolopendracataracta* Siriwut, Edgecombe & Panha, 2016 that entered a stream to escape from the collector, and the same behaviour was observed by one of the authors (I.H. Tuf pers. obs.) in *Lithobiusforficatus* (Linnaeus, 1758) and *Lithobiusmutabilis* L. Koch, 1862.

Probably the most frequent reasons for myriapods to come into contact with water are tides and floods. From tide-affected seashores there are numerous reports of more than 40 centipede taxa (see review by Barber 2009, 2011). Almost all of these are geophilomorphs, many of which are considered as real halophiles with adaptations to submergence (Binyon and Lewis 1963; Barber 2011), while the recorded ubiquitous lithobiomorph species do not appear to be truly halophilic; the only exception might be *Lithobiusellipticus* Takakuwa, 1939 (Barber 2009). Several millipede species are able to survive river floods by living actively under floodwaters for weeks (Golovatch and Kime 2009), while some centipedes have dormant submerged egg stage (e.g. *Lamyctesadisi* Zalesskaja, 1994 (Zalesskaja 1994) and *Lamyctesemarginatus* (Zulka 1991; Zerm 1997)). The centipede *Lithobiuscurtipes* is able to survive under water for more than one week under experimental conditions (Tufová and Tuf 2005), while in an experiment *Scolopendrasubspinipes* Leach, 1816 was found to swim on the water surface, probably as a strategy for escape during floods (Lewis 1980). Another scolopendromorph, *Edentistomaoctosulcatum* Tömösváry, 1882, does not swim, but in an experiment by Lewis (1980) simply walked along the bottom when inundated. *L.matulici* and related species inhabit caves where flash floods are common and which have active streams of highly fluctuating water levels, depending on the precipitation and/or snow melting at the surface region above them (Spahić 2015). In addition to the necessity of tolerating submergence during floods, the ability to submerge voluntarily and to move under water might be also useful in moving between parts of the cave that are separated by water. Semiaquatic behaviour might be potentially highly adaptive in caves also for another reason: in subterranean habitats food sources are limited and an expansion of the prey spectrum with the inclusion of the aquatic biota can help a terrestrial predator increase its fitness. This might be highly significant, especially when aquatic biota (e.g. *Niphargus* amphipods) represents the main part of the available biomass of possible prey, like in the caves discussed here (Gergely Balázs pers. obs.; Márton Mede in litt.). Due to similar conditions and forces, such adaptation might be hypothesized to emerge in parallel multiple times in different caves, just like in some hydrophilous millipede taxa (Enghoff 1985).

**Two other cave-dwelling *Lithobius* (s.s.) species from the Dinaric Mountains.***Lithobiussketi* Matic & Dărăbanţu, 1968 was described as belonging to the subgenus Troglolithobius Matic, 1967 (junior synonym of *Lithobius* according to Stoev 1997), which included also *L.matulici* at that time, and it was stated to be very similar to that species. Although no rounded edge of tergite 14 is known for *L.sketi*, additional studies are needed to verify this character in this species. *Lithobiustroglomontanus* (Folkmanová, 1940) was described from Vodna Cave (Vodna Pećina), Montenegro, but it is missing from the list of Mitić et al. (2007). Although Kos (1992) considered *L.troglomontanus* closely related to *L.matulici* and *L.sketi* and also as belonging to the subgenus Troglolithobius Matic, 1967, it seems to be different indeed from *L.matulici* in the shape of tergite 14 (with cornered posterior edges). It shares several characters with *L.sketi*, i.e. the structure of female gonopods (unipartite gonopodal claws, 1+1 spurs) and the arrangement of the coxal pores (smaller pores forming a second row), but they do differ in several important characters. Some of these differences (small Tömösváry’s organ, short ultimate legs and antennae) actually show *troglomontanus* to be morphologically not very cave-adapted, and thus, surface collecting around the type locality cave might prove it to be not a real troglobiont species. Based on the differences, we consider *L.troglomontanus* and *L.sketi* to be two valid species, but molecular phylogenetic studies are needed on each of these cave taxa to clarify their actual relation to each other and to the members of other subgenera within *Lithobius*.

## Supplementary Material

XML Treatment for Lithobius (Lithobius) matulici

## Supplementary materials

Supplementary material 1 Lithobiomorph specimen under water in the Tibia-Fresca Cave System (North Spain) (video by Zsolt Polacsek) Data type: multimedia This dataset is made available under the Open Database License (http://opendatacommons.org/licenses/odbl/1.0/). The Open Database License (ODbL) is a license agreement intended to allow users to freely share, modify, and use this Dataset while maintaining this same freedom for others, provided that the original source and author(s) are credited.László Dányi, Gergely Balázs, Ivan Hadrián Tuf
